# Poly[μ_2_-aqua-aqua­[μ_3_-*N*-butyl-*N*-(2-hy­droxy­ethyl)di­thio­carbamato-κ^3^ *O*,*O*′:*S*]sodium]

**DOI:** 10.1107/S2056989016000657

**Published:** 2016-01-20

**Authors:** Muzzaffar A. Bhat, Shalini Jain, Sanjay K. Srivastava, Ray J. Butcher, Jan Wikaira

**Affiliations:** a School of Studies in Chemistry, Jiwaji University, Gwalior 474 011, India; b Department of Chemistry, Howard University, 525 College Street NW, Washington, DC 20059, USA; c Department of Chemistry, University of Canterbury, Private Bag 4800, Christchurch, New Zealand

**Keywords:** crystal structure, di­thio­carbamate, sodium salt, two-dimensional polymeric structure

## Abstract

In the title compound, [Na(C_7_H_14_NOS_2_)(H_2_O)_2_]_*n*_, the Na^I^ cation is coordinated by five O atoms [Na—O = 2.3142 (11)–2.4677 (10) Å] from three aqua and two *N*-butyl-*N*-(2-hy­droxy­eth­yl)di­thio­carbamate (*L*) ligands and one S atom [Na—S = 3.0074 (6) Å] from a third *L* ligand in a highly distorted octa­hedral geometry. Two aqua ligands related by an inversion center bridge two Na^I^ cations, and each *L* ligand coordinates three Na^I^ cations, leading to a layered arrangement aligned parallel to the *bc* plane. Inter­molecular O—H⋯S hydrogen bonds are observed in the inner part of each polymeric layer; these are packed along the *a* axis and held together by weak van der Waals forces.

## Abstract 2

The title mol­ecule with empirical formula, [Na(μ_3_-C_6_H_14_ONCS_2_)(μ_2_-H_2_O)(H_2_O)], contains a triply bridging *N*-butyl-*N*-(2-hy­droxy­eth­yl)di­thio­carbamate anion and forms a two-dimensional polymer.

## Chemical context   

Di­thio­carbamates have recently drawn more attention due to their application in group-transfer radical cyclization reactions (Grainger & Innocenti, 2007 ▸) and as ligands for chelating metals (Greenwood & Earnshaw, 1997 ▸). In recent years, their applications have not only become apparent as pesticides and fungicides, but they have also been widely used as vulcanization accelerators in the rubber industry (Svetlik *et al.*, 1955 ▸). Di­thio­carbamates are also of biological importance due to their anti­cancer, anti­bacterial, anti­tuberculosis and anti­fungal properties (Li *et al.*, 2015 ▸; Sim *et al.*, 2014 ▸; Chauhan *et al.*, 2012 ▸; Byrne *et al.*, 2007 ▸). Their anti-oxidant properties make them even more valuable compounds. As part of our investigations on organotindi­thio complexes (Srivastava *et al.*, 2007 ▸), we herein report the synthesis and structure of the title compound.
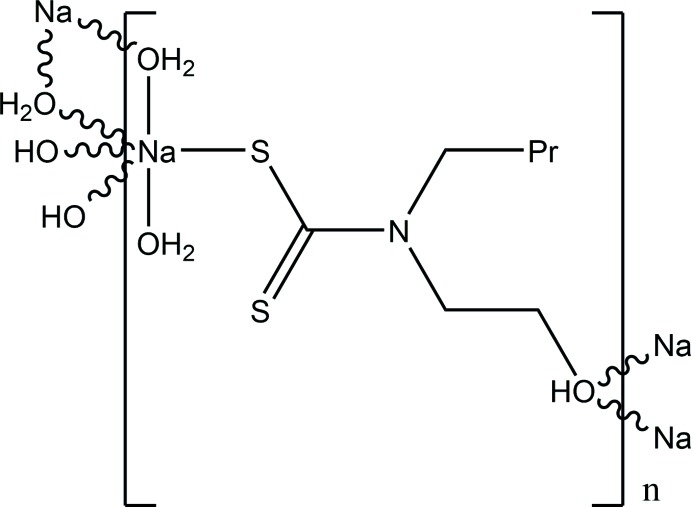


## Structural commentary   

**Figure 1:**
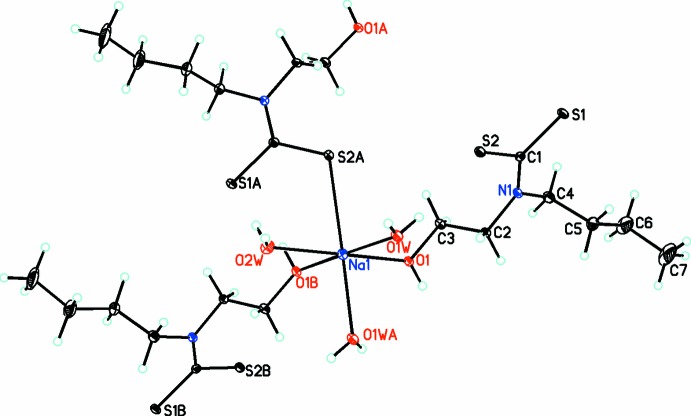
A portion of the title crystal structure showing the coordination environment for the Na^I^ cation and the atomic labels [symmetry codes: (A) 1 − *x*, *y* − 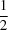, 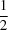 − *z*; (B) 1 − *x*, −*y*, 1 − *z*]. Displacement ellipsoids are drawn at the 30% probability level.

**Figure 2:**
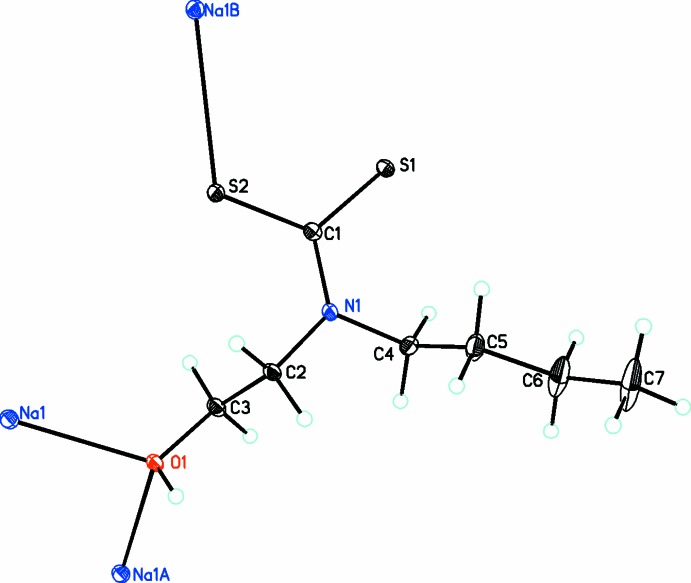
Diagram showing the triply bridging nature of the di­thio­carbamate anion [symmetry codes: (A) 1 − *x*, −*y*, 1 − *z*; (B) 1 − *x*, *y* + 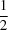, 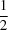 − *z*].

The title compound is a two-dimensional polymer with formula [Na(μ_3_-C_7_H_14_NOS_2_)(μ_2_-H_2_O)(H_2_O)]. Within this polymer, each Na^I^ ion exhibits a distorted octa­hedral geometry (Fig. 1 ▸) made up from coordination by the S atom of one *N*-butyl-*N*-(2-hy­droxy­eth­yl)di­thio­carbamate (*L*) anion, two hy­droxy O atoms from two *L* ligands and three aqua ligands, of which two aqua ligands form bridging units between two Na^I^ cations. The di­thio­carbamate anion acts as a triply bridging ligand, where one S atom coordinates one sodium atom and the O_hy­droxy_ atom coordinates two sodium atoms (Fig. 2 ▸). The aforementioned feature of multiple coord­ination modes leads to the formation of polymeric layers parallel to the *bc* plane with the hydro­phobic butyl arms protruding up and down. In the *L* ligand, while the two S atoms are not chemically equivalent as only one is involved in bonding to the Na cation, the C—S bond lengths are identical at 1.726 (1) Å.

## Supra­molecular features   

**Figure 3:**
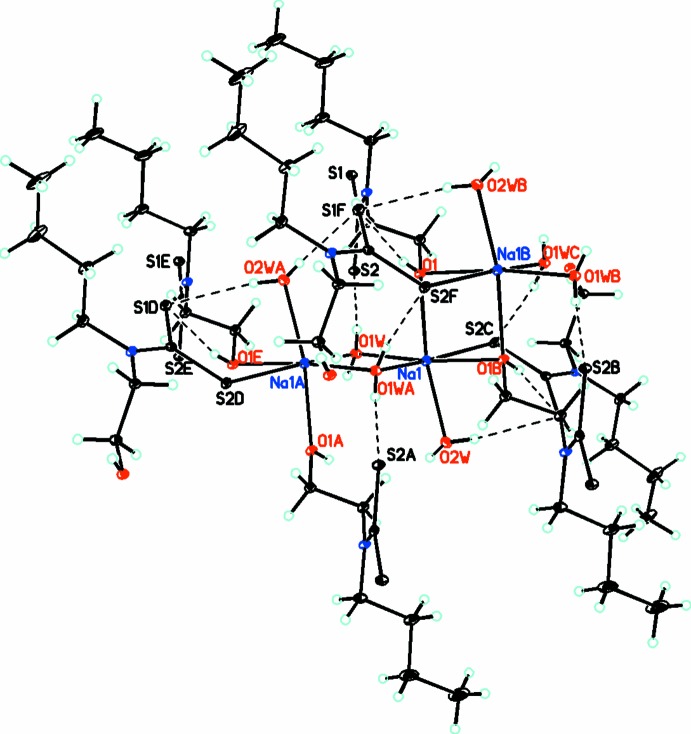
A portion of the crystal packing showing the O—H⋯S hydrogen bonds (dashed lines) in the inner part of the polymeric layer [symmetry codes: (A) 1 − *x*, 1 − *y*, 1 − *z*; (B) 1 − *x*, *y* − 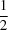, 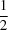 − *z*; (C) 1 − *x*, −*y*, 1 − *z*; (D) *x*, 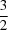 − *y*, 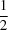 + *z*; (E) *x*, 1 + *y*, *z*; (F) *x*, 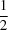 − *y*, 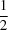 + *x*].

Inter­molecular O—H⋯S hydrogen bonds (Table 1 ▸) are observed in the inner part of each polymeric layer (Fig. 3 ▸). The layers are further packed along the *a* axis and held together by weak van der Waals forces.

## Database survey   

In a recent publication, Howie *et al.* (2008 ▸) reported a structurally similar compound where the butyl substituent was replaced by a propyl substituent. The crystal structures of other sodium salts of di­thio­carbamates, Na[S_2_CN(C_2_H_5_)_2_]·3H_2_O (Colapietro *et al.*, 1968 ▸), Na[S_2_CN(CH_2_)_4_]·2H_2_O (Albertsson *et al.*, 1980 ▸; Ymén, 1982 ▸), Na[S_2_CN(C_3_H_7_)_2_]·5H_2_O (Ymén, 1983 ▸) and Na[S_2_CN(CH_3_)_2_]·2H_2_O (Oskarsson & Ymén, 1983 ▸), Na[S_2_CN(CH_2_)_5_]·2H_2_O (Mafud & Gambardella, 2011 ▸), Na[S_2_CN(C_8_H_5_NS)]·3H_2_O (Téllez *et al.*, 2004 ▸) have been reported. All these structures are polymeric in nature and contain the μ(H_2_O)_2_Na_2_ unit.

## Synthesis and crystallization   

The title compound was prepared by the reaction of *N*-butyl *N*-hy­droxy­ethyl amine (0.01 mol), carbon di­sulfide (0.01 mol) and sodium hydroxide (0.01 mol) in dry diethyl ether and was stirred for 4 h at 253 K. The crude product was recrystallized from isopropyl alcohol. It was then dissolved in a hexa­ne:diethyl ether (1:1 *v*/*v*) mixture and put in a deep freezer overnight. Square transparent crystals suitable for X ray analysis were obtained in 80% yield (m.p.: 430 K). Analysis calculated for C_7_H_18_NO_3_S_2_ (%) S, 29.78; found: S, 29.84.

## Refinement   

Crystal data, data collection and structure refinement details are summarized in Table 2 ▸. All C-bound H atoms were idealized with C—H distances of 0.99 Å for CH_2_ and 0.98 Å for CH_3_ groups with atomic displacement parameters of *U_iso_*(H) = 1.5*U*_eq_(C) for methyl H atoms and 1.2*U_eq_*(C) for other H atoms. The water and hydroxyl H atoms were freely refined.

## Supplementary Material

Crystal structure: contains datablock(s) I. DOI: 10.1107/S2056989016000657/cv5500sup1.cif

Structure factors: contains datablock(s) I. DOI: 10.1107/S2056989016000657/cv5500Isup2.hkl

CCDC reference: 1447132

Additional supporting information:  crystallographic information; 3D view; checkCIF report

## Figures and Tables

**Table 1:** Hydrogen-bond geometry (Å, °)

*D*—H⋯*A*	*D*—H	H⋯*A*	*D*⋯*A*	*D*—H⋯*A*
O1—H1*O*⋯S1^i^	0.82 (2)	2.41 (2)	3.2227 (10)	167.6 (19)
O1*W*—H1*W*1⋯S2	0.82 (2)	2.48 (2)	3.2933 (10)	168 (2)
O1*W*—H1*W*2⋯S2^ii^	0.85 (3)	2.42 (3)	3.2605 (10)	171 (2)
O2*W*—H2*W*2⋯S1^ii^	0.78 (2)	2.48 (2)	3.2624 (11)	173 (2)

Symmetry codes: (i) 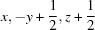; (ii) 
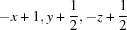
.

**Table 2:** Experimental details

Crystal data	
Chemical formula	[Na(C_7_H_14_NOS_2_)(H_2_O)_2_]
*M*_r_	251.33
Crystal system, space group	Monoclinic, *P*2_1_/*c*
Temperature (K)	120
*a*, *b*, *c* (Å)	15.6223 (3), 5.8379 (1), 14.7114 (3)
β (°)	101.868 (2)
*V* (Å^3^)	1313.02 (4)
*Z*	4
Radiation type	Cu *K*α
μ (mm^−1^)	3.90
Crystal size (mm)	0.39 × 0.31 × 0.24

Data collection	
Diffractometer	Agilent SuperNova Dual Source diffractometer with an Atlas detector
Absorption correction	Multi-scan (*CrysAlis PRO*; Agilent, 2012 ▸)
*T*_min_, *T*_max_	0.660, 1.000
No. of measured, independent and observed [*I* > 2σ(*I*)] reflections	5973, 2731, 2617
*R*_int_	0.019
(sin θ/λ)_max_ (Å^−1^)	0.631

Refinement	
*R*[*F* ^2^ > 2σ(*F* ^2^)], *wR*(*F* ^2^), *S*	0.029, 0.080, 1.05
No. of reflections	2731
No. of parameters	149
H-atom treatment	H atoms treated by a mixture of independent and constrained refinement
Δρ_max_, Δρ_min_ (e Å^−3^)	0.29, −0.39

Computer programs: *CrysAlis PRO* (Agilent, 2012 ▸), *SHELXTL* (Sheldrick, 2008 ▸) and *SHELXL2014* (Sheldrick, 2015 ▸).

## supplementary crystallographic information

### Crystal data

**Table:**

[Na(C_7_H_14_NOS_2_)(H_2_O)_2_]	*F*(000) = 536
*M**_r_* = 251.33	*D*_x_ = 1.271 Mg m^−^^3^
Monoclinic, *P*2_1_/*c*	Cu *K*α radiation, λ = 1.54184 Å
*a* = 15.6223 (3) Å	Cell parameters from 4003 reflections
*b* = 5.8379 (1) Å	θ = 3.1–76.6°
*c* = 14.7114 (3) Å	µ = 3.90 mm^−^^1^
β = 101.868 (2)°	*T* = 120 K
*V* = 1313.02 (4) Å^3^	Chunk, colorless
*Z* = 4	0.39 × 0.31 × 0.24 mm

### Data collection

**Table:**

Agilent SuperNova Dual Source diffractometer with an Atlas detector	2731 independent reflections
Radiation source: sealed X-ray tube	2617 reflections with *I* > 2σ(*I*)
Detector resolution: 10.6501 pixels mm^-1^	*R*_int_ = 0.019
ω scans	θ_max_ = 76.4°, θ_min_ = 2.9°
Absorption correction: multi-scan (*CrysAlis PRO*; Agilent, 2012)	*h* = −19→17
*T*_min_ = 0.660, *T*_max_ = 1.000	*k* = −7→4
5973 measured reflections	*l* = −18→17

### Refinement

**Table:**

Refinement on *F*^2^	Hydrogen site location: mixed
Least-squares matrix: full	H atoms treated by a mixture of independent and constrained refinement
*R*[*F*^2^ > 2σ(*F*^2^)] = 0.029	*w* = 1/[σ^2^(*F*_o_^2^) + (0.0516*P*)^2^ + 0.3898*P*] where *P* = (*F*_o_^2^ + 2*F*_c_^2^)/3
*wR*(*F*^2^) = 0.080	(Δ/σ)_max_ = 0.001
*S* = 1.05	Δρ_max_ = 0.29 e Å^−^^3^
2731 reflections	Δρ_min_ = −0.39 e Å^−^^3^
149 parameters	Extinction correction: *SHELXL2014* (Sheldrick, 2008), Fc^*^=kFc[1+0.001xFc^2^λ^3^/sin(2θ)]^-1/4^
0 restraints	Extinction coefficient: 0.0041 (4)

### Special details

**Table:**

Geometry. All e.s.d.'s (except the e.s.d. in the dihedral angle between two l.s. planes) are estimated using the full covariance matrix. The cell e.s.d.'s are taken into account individually in the estimation of e.s.d.'s in distances, angles and torsion angles; correlations between e.s.d.'s in cell parameters are only used when they are defined by crystal symmetry. An approximate (isotropic) treatment of cell e.s.d.'s is used for estimating e.s.d.'s involving l.s. planes.

### Fractional atomic coordinates and isotropic or equivalent isotropic displacement parameters (Å^2^)

**Table:**

	*x*	*y*	*z*	*U*_iso_*/*U*_eq_	
Na1	0.44835 (3)	0.26004 (8)	0.45654 (3)	0.01595 (14)	
S1	0.76058 (2)	0.34854 (5)	0.14289 (2)	0.01645 (11)	
S2	0.60961 (2)	0.50629 (5)	0.22351 (2)	0.01569 (11)	
O1	0.59309 (6)	0.09872 (16)	0.47496 (6)	0.0155 (2)	
H1O	0.6306 (14)	0.125 (4)	0.5218 (15)	0.035 (5)*	
O1W	0.50061 (6)	0.58495 (17)	0.39025 (6)	0.0172 (2)	
H1W1	0.5325 (15)	0.551 (4)	0.3542 (15)	0.039 (6)*	
H1W2	0.4670 (16)	0.686 (4)	0.3609 (16)	0.046 (6)*	
O2W	0.30199 (7)	0.34773 (19)	0.44462 (7)	0.0220 (2)	
H2W1	0.2792 (15)	0.233 (4)	0.4121 (16)	0.041 (6)*	
H2W2	0.2843 (15)	0.462 (4)	0.4197 (15)	0.036 (6)*	
N1	0.73937 (7)	0.23989 (18)	0.31192 (7)	0.0149 (2)	
C1	0.70580 (8)	0.3542 (2)	0.23350 (8)	0.0138 (2)	
C2	0.70139 (8)	0.2560 (2)	0.39489 (8)	0.0154 (3)	
H2A	0.6735	0.4079	0.3964	0.019*	
H2B	0.7485	0.2424	0.4510	0.019*	
C3	0.63355 (8)	0.0692 (2)	0.39629 (8)	0.0167 (3)	
H3A	0.5884	0.0758	0.3383	0.020*	
H3B	0.6621	−0.0828	0.3996	0.020*	
C4	0.82101 (9)	0.1072 (2)	0.32374 (9)	0.0197 (3)	
H4A	0.8270	0.0440	0.2629	0.024*	
H4B	0.8184	−0.0229	0.3662	0.024*	
C5	0.90068 (9)	0.2546 (3)	0.36299 (10)	0.0269 (3)	
H5A	0.9006	0.3906	0.3228	0.032*	
H5B	0.8963	0.3091	0.4256	0.032*	
C6	0.98645 (11)	0.1266 (4)	0.36977 (15)	0.0465 (5)	
H6A	0.9929	0.0811	0.3067	0.056*	
H6B	0.9852	−0.0147	0.4067	0.056*	
C7	1.06488 (12)	0.2721 (5)	0.41491 (19)	0.0658 (8)	
H7A	1.1185	0.1809	0.4213	0.099*	
H7B	1.0576	0.3224	0.4764	0.099*	
H7C	1.0689	0.4063	0.3760	0.099*	

### Atomic displacement parameters (Å^2^)

**Table:**

	*U*^11^	*U*^22^	*U*^33^	*U*^12^	*U*^13^	*U*^23^
Na1	0.0155 (3)	0.0175 (3)	0.0152 (2)	−0.00088 (18)	0.00403 (19)	0.00032 (18)
S1	0.01710 (17)	0.02187 (18)	0.01177 (17)	0.00208 (11)	0.00623 (12)	0.00148 (10)
S2	0.01414 (17)	0.02106 (18)	0.01238 (17)	0.00310 (10)	0.00394 (11)	0.00173 (10)
O1	0.0133 (4)	0.0226 (5)	0.0114 (4)	−0.0007 (3)	0.0044 (3)	0.0013 (3)
O1W	0.0175 (4)	0.0200 (5)	0.0152 (4)	0.0025 (4)	0.0061 (4)	0.0011 (4)
O2W	0.0203 (5)	0.0190 (5)	0.0257 (5)	0.0024 (4)	0.0024 (4)	−0.0033 (4)
N1	0.0121 (5)	0.0214 (5)	0.0118 (5)	0.0007 (4)	0.0040 (4)	0.0013 (4)
C1	0.0136 (6)	0.0157 (6)	0.0118 (5)	−0.0035 (4)	0.0020 (4)	−0.0014 (4)
C2	0.0141 (6)	0.0226 (6)	0.0100 (5)	−0.0014 (5)	0.0036 (4)	0.0014 (4)
C3	0.0174 (6)	0.0211 (6)	0.0130 (6)	−0.0013 (5)	0.0062 (4)	−0.0006 (5)
C4	0.0166 (6)	0.0260 (6)	0.0167 (6)	0.0060 (5)	0.0038 (5)	0.0034 (5)
C5	0.0143 (7)	0.0410 (9)	0.0250 (7)	0.0026 (6)	0.0027 (5)	−0.0022 (6)
C6	0.0172 (8)	0.0670 (13)	0.0528 (11)	0.0100 (8)	0.0015 (7)	−0.0184 (10)
C7	0.0139 (8)	0.0942 (19)	0.0856 (18)	0.0041 (10)	0.0015 (9)	−0.0321 (15)

### Geometric parameters (Å, º)

**Table:**

Na1—O2W	2.3142 (11)	N1—C1	1.3425 (16)
Na1—O1W	2.3544 (11)	N1—C2	1.4658 (15)
Na1—O1W^i^	2.4074 (11)	N1—C4	1.4717 (16)
Na1—O1	2.4128 (10)	C2—C3	1.5237 (17)
Na1—O1^ii^	2.4677 (10)	C2—H2A	0.9900
Na1—S2^iii^	3.0074 (6)	C2—H2B	0.9900
Na1—Na1^i^	3.3527 (10)	C3—H3A	0.9900
Na1—Na1^ii^	3.5509 (10)	C3—H3B	0.9900
Na1—H2W1	2.59 (2)	C4—C5	1.525 (2)
S1—C1	1.7261 (13)	C4—H4A	0.9900
S2—C1	1.7256 (13)	C4—H4B	0.9900
S2—Na1^iv^	3.0073 (6)	C5—C6	1.519 (2)
O1—C3	1.4386 (14)	C5—H5A	0.9900
O1—Na1^ii^	2.4677 (10)	C5—H5B	0.9900
O1—H1O	0.82 (2)	C6—C7	1.526 (3)
O1W—Na1^i^	2.4074 (10)	C6—H6A	0.9900
O1W—H1W1	0.82 (2)	C6—H6B	0.9900
O1W—H1W2	0.85 (3)	C7—H7A	0.9800
O2W—H2W1	0.86 (2)	C7—H7B	0.9800
O2W—H2W2	0.78 (2)	C7—H7C	0.9800
			
O2W—Na1—O1W	102.22 (4)	Na1^i^—O1W—H1W2	105.5 (16)
O2W—Na1—O1W^i^	96.88 (4)	H1W1—O1W—H1W2	103 (2)
O1W—Na1—O1W^i^	90.49 (4)	Na1—O2W—H2W1	99.2 (15)
O2W—Na1—O1	169.54 (4)	Na1—O2W—H2W2	118.2 (16)
O1W—Na1—O1	87.95 (4)	H2W1—O2W—H2W2	110 (2)
O1W^i^—Na1—O1	85.37 (4)	C1—N1—C2	122.02 (10)
O2W—Na1—O1^ii^	83.15 (4)	C1—N1—C4	122.61 (11)
O1W—Na1—O1^ii^	174.49 (4)	C2—N1—C4	115.15 (10)
O1W^i^—Na1—O1^ii^	90.05 (4)	N1—C1—S2	120.56 (9)
O1—Na1—O1^ii^	86.64 (3)	N1—C1—S1	119.09 (9)
O2W—Na1—S2^iii^	85.90 (3)	S2—C1—S1	120.36 (7)
O1W—Na1—S2^iii^	95.69 (3)	N1—C2—C3	111.54 (10)
O1W^i^—Na1—S2^iii^	172.54 (3)	N1—C2—H2A	109.3
O1—Na1—S2^iii^	90.69 (3)	C3—C2—H2A	109.3
O1^ii^—Na1—S2^iii^	83.39 (3)	N1—C2—H2B	109.3
O2W—Na1—Na1^i^	103.57 (4)	C3—C2—H2B	109.3
O1W—Na1—Na1^i^	45.89 (3)	H2A—C2—H2B	108.0
O1W^i^—Na1—Na1^i^	44.60 (3)	O1—C3—C2	110.32 (10)
O1—Na1—Na1^i^	85.23 (3)	O1—C3—H3A	109.6
O1^ii^—Na1—Na1^i^	134.40 (3)	C2—C3—H3A	109.6
S2^iii^—Na1—Na1^i^	141.41 (3)	O1—C3—H3B	109.6
O2W—Na1—Na1^ii^	125.82 (4)	C2—C3—H3B	109.6
O1W—Na1—Na1^ii^	131.86 (3)	H3A—C3—H3B	108.1
O1W^i^—Na1—Na1^ii^	86.89 (3)	N1—C4—C5	111.56 (12)
O1—Na1—Na1^ii^	43.93 (2)	N1—C4—H4A	109.3
O1^ii^—Na1—Na1^ii^	42.71 (2)	C5—C4—H4A	109.3
S2^iii^—Na1—Na1^ii^	85.882 (19)	N1—C4—H4B	109.3
Na1^i^—Na1—Na1^ii^	115.45 (3)	C5—C4—H4B	109.3
O2W—Na1—H2W1	19.0 (5)	H4A—C4—H4B	108.0
O1W—Na1—H2W1	111.5 (5)	C6—C5—C4	112.80 (14)
O1W^i^—Na1—H2W1	112.5 (5)	C6—C5—H5A	109.0
O1—Na1—H2W1	152.6 (6)	C4—C5—H5A	109.0
O1^ii^—Na1—H2W1	73.3 (5)	C6—C5—H5B	109.0
S2^iii^—Na1—H2W1	69.0 (5)	C4—C5—H5B	109.0
Na1^i^—Na1—H2W1	122.2 (5)	H5A—C5—H5B	107.8
Na1^ii^—Na1—H2W1	113.8 (5)	C5—C6—C7	111.93 (18)
C1—S2—Na1^iv^	115.32 (4)	C5—C6—H6A	109.2
C3—O1—Na1	120.87 (7)	C7—C6—H6A	109.2
C3—O1—Na1^ii^	115.00 (8)	C5—C6—H6B	109.2
Na1—O1—Na1^ii^	93.36 (3)	C7—C6—H6B	109.2
C3—O1—H1O	109.9 (14)	H6A—C6—H6B	107.9
Na1—O1—H1O	120.7 (14)	C6—C7—H7A	109.5
Na1^ii^—O1—H1O	91.1 (15)	C6—C7—H7B	109.5
Na1—O1W—Na1^i^	89.50 (4)	H7A—C7—H7B	109.5
Na1—O1W—H1W1	112.5 (16)	C6—C7—H7C	109.5
Na1^i^—O1W—H1W1	124.5 (15)	H7A—C7—H7C	109.5
Na1—O1W—H1W2	122.7 (16)	H7B—C7—H7C	109.5
			
C2—N1—C1—S2	−5.92 (16)	Na1—O1—C3—C2	102.88 (10)
C4—N1—C1—S2	179.80 (9)	Na1^ii^—O1—C3—C2	−146.38 (8)
C2—N1—C1—S1	173.66 (9)	N1—C2—C3—O1	−176.02 (9)
C4—N1—C1—S1	−0.62 (16)	C1—N1—C4—C5	89.35 (15)
Na1^iv^—S2—C1—N1	178.00 (8)	C2—N1—C4—C5	−85.29 (13)
Na1^iv^—S2—C1—S1	−1.57 (9)	N1—C4—C5—C6	−176.06 (13)
C1—N1—C2—C3	91.65 (14)	C4—C5—C6—C7	−176.34 (18)
C4—N1—C2—C3	−93.68 (13)		

Symmetry codes: (i) −*x*+1, −*y*+1, −*z*+1; (ii) −*x*+1, −*y*, −*z*+1; (iii) −*x*+1, *y*−1/2, −*z*+1/2; (iv) −*x*+1, *y*+1/2, −*z*+1/2.

### Hydrogen-bond geometry (Å, º)

**Table:**

*D*—H···*A*	*D*—H	H···*A*	*D*···*A*	*D*—H···*A*
O1—H1*O*···S1^v^	0.82 (2)	2.41 (2)	3.2227 (10)	167.6 (19)
O1*W*—H1*W*1···S2	0.82 (2)	2.48 (2)	3.2933 (10)	168 (2)
O1*W*—H1*W*2···S2^iv^	0.85 (3)	2.42 (3)	3.2605 (10)	171 (2)
O2*W*—H2*W*2···S1^iv^	0.78 (2)	2.48 (2)	3.2624 (11)	173 (2)

Symmetry codes: (iv) −*x*+1, *y*+1/2, −*z*+1/2; (v) *x*, −*y*+1/2, *z*+1/2.
